# Cultures of Activity, Cultivating Resistance

**DOI:** 10.1177/10497323241271915

**Published:** 2024-10-23

**Authors:** Katherine Kenny, Alex Broom, Michelle Peterie, Juliet Bennett, Jennifer Broom

**Affiliations:** 1 Sydney Centre for Healthy Societies, The School of Social and Political Sciences, Faculty of Arts and Social Sciences, 4334 The University of Sydney, Sydney, NSW, Australia; 2 Sydney Centre for Healthy Societies, School of Social and Political Sciences, Faculty of Arts and Social Sciences, and Charles Perkins Centre, 4334 The University of Sydney, Sydney, NSW, Australia; 3 Medical School (GBCS), Faculty of Medicine, 1974 The University of Queensland, Brisbane, QLD, Australia

**Keywords:** antimicrobial resistance, activity-based funding, hospital governance, antimicrobial stewardship, qualitative

## Abstract

The problem of antimicrobial resistance (AMR) is often viewed through biomedical and/or behavioral lenses, with the underlying economic principles and “headwinds” shaping resistance less visible. In this paper, we focus on how healthcare funding models structure the ways AMR is perceived and addressed as an institutional priority. Specifically, we explore how activity-based funding reflects and operationalizes ingrained assumptions about what is valuable and/or worthwhile within the organizational ecology of the hospital. Drawing on interviews with 36 executives from several hospital clinical care settings across two Australian states, we illuminate the ways the activity-based funding paradigm works against efforts to combat AMR. Concerningly, we further observe how activity-based funding models can inadvertently position rising rates of resistance as a benefit—at least in the short term—as the new and intensified interventions required to address resistant infections require more “activity” and thus deliver higher reimbursement at the level of annualized budgets. In failing to recognize the (social and economic) value of *reduced* activity, activity-based funding risks contributing to AMR, rather than working to resolve it.

## Introduction

*Antimicrobial resistance* (AMR), where bacteria or other microbes develop resistance to the medications that once killed them, has recently been identified as one of the 10 greatest threats to global health (World Health Organization [WHO], 2021). Ranging from increased risk associated with previously basic surgical procedures, to longer hospital stays associated with complications and resistant infections, to exacerbated risk to immune-compromised patients, the consequences of rising rates of AMR are serious, ubiquitous, and accelerating (Australian Commission on Safety and Quality in Health Care [ACSQHC], 2021; Broom, Kenny, Prainsack, et al., 2021; Holmes et al., 2016; Laxminarayan, 2022). Nevertheless, widespread (over)prescribing of antimicrobials continues almost unabated (see Broom et al., 2014, 2017; Broom, Broom, et al., 2016; Broom, Gibson, et al., 2016; Broom, Kenny, Kirby, et al., 2021; Broom, Kenny, Prainsack, et al., 2021; Broom, Peterie, Kenny, Ramia, et al., 2023). *Antimicrobial stewardship* (AMS) activities—behavioral change programs aimed at curtailing excess prescribing—often lack local institutional buy-in and/or adequate financing (Gray, 2017) and can even jar with dominant models of funding and governance in the hospital setting (see Broom, Kenny, Kirby, et al., 2021).

Since the 1990s, activity-based funding has gained considerable traction and is now the most commonly used model for funding acute care internationally (Baxter et al., 2015). *Activity-based funding* is a method of calculating hospital funding based on the volume and nature of health services delivered to patients of similar diagnosis. As illustrated in various studies of activity-based funding in Europe (O’Reilly et al., 2012) and North America (Sutherland et al., 2012), activity-based funding has become the gold standard for maximizing activity and efficiency, incentivizing hospitals to treat more patients, faster. While providing a transparent, centralized tool for funding hospitals, it has also faced substantial critique, generally for “its emphasis on volumes and reducing costs rather than the provision of high-quality hospital care” (Baxter et al., 2015, p. 1097). *Activity*—the delivery of discrete services to individual patients—is now a primary logic of accounting for, financing, and prioritizing particular initiatives in the healthcare system. Augmented by accreditation processes and the implementation of national standards, such modes of organizing healthcare were intended to increase transparency in healthcare funding, simplifying what is counted and amounts paid for different healthcare provisions. This was a means to incentivize efficiencies within service provision and to standardize care across health districts, states, and the nation (Solomon, 2014). These attempts at efficiency and standardization, underpinned by concrete deliverables (“activities”), enabled the widespread application of cost–benefit analyses in the administration and governance of healthcare. However, this approach has not adequately accounted for the question of *value* beyond mere economic cost. Funding healthcare systems through activity tied to annualized budget cycles inevitably disincentivizes the provision of care that transcends the individual patient or budget-years. For example, community-oriented public health initiatives or long-term investments in areas such as preventative care are not part of this model because, as Hemenway (2010) points out, public health investments often yield benefits that are only obvious over the mid- to long term, have beneficiaries that are largely anonymous, are driven by benefactors that rarely receive public recognition, and often require societal changes and disruptions to the status quo that are notoriously difficult to achieve politically. Prevention of illness and disease, in effect, remains the poor cousin of “real” medical intervention administered to individuals (Galea & Annas, 2016; Hemenway, 2021).

While the specificities of healthcare financing most directly pertain to the economic costs of service provision, they unavoidably also shape what is considered *of value* within institutions, what is prioritized, and what is implemented (cf. Gray, 2017). How much funding is available, who and what attracts funding, and the logics that determine resource allocation thus all demand careful sociological consideration (cf. Mossialos & Dixon, 2002). In this paper, drawing on interviews with healthcare executives across two Australian states and focusing the case of AMR, we examine the interconnectedness between health threats, their potential solutions, and the ways that institutional financing systems variously enable and/or constrain what is possible.

### Activity-Based Funding in the Australian Healthcare System

In Australia, healthcare is provided through a dual system of publicly and privately funded services. Community-based primary care (by general practitioners) and public secondary and tertiary care (highly specialized care in hospitals) are publicly funded. These sit alongside private secondary and tertiary care (specialists), including some (often “elective”) inpatient care, which are privately funded. Although primary care services (general practitioners) receive public funding, they frequently require patient co-payments (Dixit & Sambasivan, 2018). Responsibility for healthcare is shared by federal and state/territory governments, with the former contributing funds and national policies, and the latter more involved in its delivery (Angeles et al., 2023).

Since the 2000s, activity-based funding has become entrenched in Australia through various national healthcare funding reforms, including the National Health Reform (NHR) Agreement. Under the NHR Agreement, activity-based funding is the primary mode of allocating financial flows from governments into the “National Health Funding Pool,” which is then distributed to local hospital networks (National Health Funding Body [NHFB], 2023b). In addition to activity-based funding for services provided, public hospitals in Australia also receive block funding in support of teaching, training and research, and public health programs. In the Australian setting, the current prevailing activity-based funding model allocates funds, in annualized blocks, based on both the volume and complexity of clinical activity. Australian-refined diagnosis-related groups (AR-DRGs) are used to classify hospital encounters and determine the exact monetary reimbursement they each “earn.”

At the institutional level, funding is managed through annualized budgets which combine *benchmarking* (estimates of a population’s size and characteristics and their anticipated healthcare needs) with *demand forecasting* (assumptions about how many interventions are likely to be performed given the size of the institution and its workforce). Mandated *limits* to annual budget increases—currently at 6.5% per annum (regardless of demand or need)—delimit the available resources, while quality is enforced through *fines* for lapses such as avoidable hospital readmissions, sentinel events, hospital-acquired complications, and other safety and quality issues (see National Health Funding Body [NHFB] 2023a for the funding formula). This funding model instils volume of activity as the primary indicator of success in assessments of value. This count of tasks completed is then also the basis for assessing whether services are meeting key performance indicators (KPIs). Implemented, as it is, alongside “new public management”–inspired efforts to extract budgetary saving within Australian health services (see Broom, Kenny, Kirby, et al., 2021), this funding model contributes to a financial habitus (cf. Bourdieu, 1990) that emphasizes efficiency and productivity within annualized budget cycles. In short, hospitals get paid for the *activities* they do within the designated reporting period.

### Attention and Accountability Under Public Sector Managerialism

As social scientists have repeatedly illustrated, forms of financial accounting and accountability—such as the Australian activity-based funding model described above—meaningfully shape institutional priorities and system-wide outcomes (Quadagno, 2010; Scambler, 2018; Schrecker, 2009). In line with international trends and the dominance of public sector managerialism more generally, the public health system in Australia has undergone considerable change in the past few decades (cf. Bennett, 2013; Briggs et al., 2012; MacIntyre, 2011). This has resulted in the emergence of throughput and finance-oriented KPIs—alongside clinical concern—to which managers and executives within the hospital setting are now beholden (see Broom, Kenny, Kirby, et al., 2021; Hood & Peters, 2004; Lyons & Dalton, 2011). In this quasi-business context, flows of funding orientate *organizational attention* (cf. Citton, 2017)—including the attention and energies of individual hospital executives—toward particular issues, shaping what is (financially) valued and prioritized and, conversely, what is sidelined, deprioritized, or delegitimized as a problem (Broom, Kenny, Kirby, et al., 2021; Broom, Kenny, Prainsack, et al., 2021; McLaughlin et al., 2002; Newman & Lawler, 2009).

The considerable research that has already been done in the social sciences around how new ideologies—neoliberal, managerial, and privatized—are inflecting healthcare systems and priorities is thus instructive (Ayo, 2012; De Vogli, 2011; Dutta, 2016). Perhaps most notably, this work shows that the reification of “activity” as a proxy for institutional success centers some practices and works against others, at times to the detriment of patient health (Dixon-Woods & Pronovost, 2016; Simonet, 2015). Modes of financing define what constitutes efficiency and productivity, attaching organizational attention to certain policies and practices while making others seem trivial or even nugatory.

### The (In)activity of Anti-Microbial Stewardship

AMR represents an instructive case of the shortcomings of prevailing activity-driven models of prioritizing and funding healthcare. Although AMR is not individually *causative* of infection, it is pervasively deteriorative. Rising AMR leads to existing infections, treatments, and activities becoming more problematic, more complex, higher risk, and more costly to treat. Efforts to counter and forestall the rise of AMR require a long-term view of activity, investment, and pay-off. Much like climate change, success is defined by a lack of *further* escalation of the problem—in this case, the proliferation of more resistance organisms. As a result, widespread agreement about the *problem* of AMR fails to yield much in the way of action. This is due, in part, to the current financing structures of hospital services.

As it stands, AMS activities do not generate fundable “activity” within the activity-based funding model. Furthermore, AMS involves a *reduction* of individualized medical intervention activities (prescribing fewer antibiotics), with longer-term shared health benefits. An important question for social science, then, is *how do the structures of healthcare financing (dis)incentivize possible AMS solutions and perpetuate the problem of AMR—in hospital settings in Australia, and beyond*?

In this paper, we explore the repercussions of these dynamics for AMR in Australian hospital contexts. More specifically, we interrogate how logics of “activity” not only determine flows of funding but also direct the attention of those involved in hospital governance in particular directions. As our participants’ accounts reveal, the moral and financial accounting “levers” associated with activity-based finding instill a desire to illustrate efficiency, to be responsible, and to “live within your means.” As we elucidate below, this creates considerable “trouble” for AMR and optimization practices therein.

## Methods

### Data Collection

This article draws on interviews with 36 hospital executives and clinical managers working in governance roles across three hospitals in two major metropolitan areas of Australia. Interviews were conducted between 2018 and 2022. Participants had a range of professional backgrounds (e.g., medical, nursing, and allied health) and were responsible for such areas in their hospitals as general management, budgets, accreditation, clinical services, departments, quality, and safety in roles such as Department and Divisional Heads, General Managers, and CEOs. Ethical approval for the study was granted through a hospital ethics committee. At each site, collaborating clinicians sent an invitation email to potential participants, after which Author 1 followed up to schedule interviews with interested parties. Working from the organizational chart of the local health district, we purposively sampled health professionals working at the level of Department Head and above in roles (a) that included hospital governance responsibilities and (b) where AMS was relevant to their day-to-day duties. All participants could be considered “hybrids” insofar as they were all (initially) clinically trained, though some were acting in roles with exclusively managerial duties (see Burgess & Currie, 2013; McGivern et al., 2015). Our focus on managerial and executive levels builds on our previous work on clinicians’ experience (Broom et al., 2014). Participants had a range of professional backgrounds (e.g., medical, nursing, and allied health) and were responsible for such areas in the hospital as general management, clinical services, patient safety, clinical improvement, emergency, infectious diseases, oncology, surgery, hematology, anesthetics, respiratory, neurology, and pharmacy. All participants provided written informed consent prior to the interview. Interviews were conducted face to face by Authors 1 and 2, both of whom are social scientists with no direct affiliation with the study sites. Each interview lasted between 30 and 80 min and was audio-recorded and later transcribed in full. Interviews focused on the day-to-day practices of governance-in-action across the full spectrum of management, including questions of priority setting, determinations of value, enacting change, managing performance, daily pressures, and short-, mid-, and long-term objectives. The focus in the interviews was on how these dimensions impacted AMR. To protect participants’ identities (particularly given the sensitive nature of some of the data), we have opted not to disclose individuals’ specific professional roles and have instead used the designation “participant” uniformly throughout.

### Data Analysis

The methodology for this project draws on the interpretive traditions within qualitative research. Data analysis was based on four questions adapted from Charmaz (1990): what is the basis of a particular experience, action, belief, relationship, or structure? What do these assume implicitly or explicitly about particular subjects and relationships? Of what larger process is this action/belief and so forth a part? What are the implications of such actions/beliefs for particular actors/institutional forms? The approach used was developmental, in that knowledge generated in the early interviews was challenged, compared with, and built upon in subsequent interviews. This approach provided an opportunity to establish initial themes and search for deviant cases, complicating our observations and retaining data complexity. We approached the analysis by conducting an initial thematic analysis, writing notes, and discussing these preliminary findings within the research team. Throughout this process, we sought to retain the richness of respondents’ experiences by documenting and discussing atypical cases, conflicts, and contradictions within the data. Once each theme was identified, we searched the transcripts for other related comments, employing constant iterative comparison to develop or complicate these themes further. This process exposed interconnections and continuities between comments and events that had initially been viewed as discrete or unrelated. The final step in the analytical process involved revisiting the literature and seeking out conceptual tools that could be employed to make sense of the patterns that were emerging from the data.

## Results

### Activity, Complexity, and Rabbit Holes

In our conversations with executives about AMR and organizational *will* to implement practice improvement around antimicrobials, a consistent theme concerned the ways prevailing funding models and allied forms of institutional accounting variously enable or foreclose solutions and improvements. Participants described the omnipotence of the (annualized) budget bottom line—calculations around which inflected all institutional decision-making—and stressed the difficulties associated with quantifying (and thus accounting for) the full costs of resistance. The opaque value of protective practices presented further challenges vis-à-vis valuing practice improvements, as it was difficult to comprehend and communicate the “real dollar” benefits of such approaches. Participant references to “real dollars” spoke to the importance of costs and benefits being *quantifiable* within the activity-driven funding scene, so that they could add value to organizational finances:

Participant: Can the metric be quantified in terms of what does [AMR] mean in terms of real dollars? I think antimicrobial resistance, the complexity of actually turning that into a dollar figure, I imagine, would require some very fancy brains. Whereas a re-presentation [of a patient to hospital]; it’s much easier to capture on an activity-based funding data collection that we already have. So, I think there’s that sort of difference, but then which one is worth more to invest in? Without looking at it [resistance], how do you know what the cost is? But then, [what if] we [are] losing money [from resistance] [later] … do we want to know how much we’re losing or wasting or added length of stay, added cost, added complexity, et cetera? […] how do you choose which one to go down the rabbit hole on?

The “rabbit hole” mentioned in the above interview refers to the seemingly endless degree of complexity that characterizes the problem of AMR. It also refers to the difficulty associated with distilling the AMS into actionable points of interventions that demonstrate “value” within the logics of (dollar based) cost–benefit analyses. Participants noted that some work was being undertaken to recognize complexity in the allocation of funding, specifically through reimbursement at higher rates for more complex care. As one manager explained, executives were increasingly encouraged to take up these funds:

Participant: I guess the other thing that I think where we need to improve on is how we document the complexity of the care we provide. So, you know that funding within New South Wales Health is based on activities […] the hospital doesn’t get the same level of funding as they do if the full complexity of the admission is reflected in the medical documentation. So that’s also, I guess, a big pressure from administration, broadly speaking, to try to get us all to reflect the complexity of what we’re doing, so that then we can attract funding that recompenses the hospital for the complexity of care we’re providing. Because if you’re somebody with community acquired pneumonia and you’ve got no other things going on, you have two or three days of IV antibiotics, go onto orals, go home, and you’re fine. That’s different to somebody with community acquired pneumonia who gets renal failure and complicated by their diabetes going out of control and this, that, and the other thing, and they require a two-week admission. If you just write the same thing on the discharge summary, you get the same amount of money, basically … On the one hand we’ve got to get people sorted quickly and get them out. On the other, we have to manage increasingly sick, complex patients and reflect that back so that the hospital gets the appropriate funding. So, I guess they’re two interesting tensions that I have.

However, in the case of AMR, this higher reimbursement rate presented a paradox, potentially rewarded rising resistance. Dealing with the complexity presented by resistant infections *increased* the resources made available at the departmental level through higher reimbursements for complex care. The benefits delivered through reductions in resistance were dismissed as too difficult to quantify:

Interviewer: … from a health service perspective, do you see the financial costs of resistance?

Participant: I don’t think that that is quantifiable in a way that an organization like this could interpret at the moment […] I don’t think we’ve got the sophistication to speak to that.

The above and other interviewees articulated the challenge of distilling diffuse and distributed contributing factors into a clear action plan, which could then be recognized as “valuable” within the driving logics of the institution. Finance, ultimately, was the opaque but powerful mediator in this process.

In particular, the complexity of not being able to identify the “real dollar” value of AMR amelioration intersected with the problems associated with improvement-as-cost. That is, in a budgetary environment where activity drives funding, improvements could produce constraints for the institution through budget reductions. As shown below, there were high-value (to the organization, as per the current funding model) practices and low-value (but high importance) practices, which meant constant organizational trade-offs between patient health and outcomes, and organizational resilience and financial stability:

Interviewer: ... when you prevent infections or when you improve in the context of antimicrobial stewardship, actually there’s less activity potentially …

Participant: Yes.

Interviewer: … if you do really well, you might have to do less.

Participant: Yeah. Yeah. The same as the program I’ve just set up called [program name] [and] rather than coming through the [hospital] department, they can call the nurse practitioner or a senior nurse and be triaged over the phone […]. But we’re losing activity from doing that program.

Interviewer: So, how do you manage that from an institutional budgetary perspective? …

Participant: … So, we need to be efficient in other areas so we can support these [non activity] programs. This is the way we look at it. Let’s get better at other things that we can control, so that we can have better patient outcomes.

Financing services on the basis of activity—to the detriment of programs that eased pressures on existing services—was a dynamic that played out even more obviously in private settings. In these contexts, activity drove not only the financing of the health service but also the salaries of private practitioners:

Participant: So if I’m in private practise and somebody comes in and … [I have] kids at private schools and got to pay the mortgage and pay the beach house and blah, blah. There is an incentive there to earn, but I’m not paid anything for trying *not* to do that, for practising in a preventative way. Everything points towards if I do *some activity*, like I put a patient in hospital, then it generates activity to the hospital, the anaesthetist has got a job, the theatre nurse has got a job.

The idea that to do good you might have to do *less* runs counter to, but is nonetheless revealing of, the incentives and norms of an organizational environment within prevailing funding structures.

### Invisible Resistance and Accounting for Success

The observation that activity-based funding necessitated different forms of (ac)counting and *making visible* recurred in the interviews. Participants observed that rising AMR was generally invisible to those in governance roles and that action often only became a priority when the problem became visible in a budgetary sense (if at all). One participant, for example, reflected on the introduction of an AMS program within their organization and the lobbying that had been done to secure institutional funds for this program. Creating a compelling business case had been core to their success:

Participant: [A colleague] was able to sell the business case to the then general manager, who came up with some funding.

Interviewer: Right. So much of this comes down to selling the business case. What are the selling points, that gain traction in the hospital?

Participant: I think he [the general manager] was looking at it [the AMS program] as an opportunity to potentially save some money. But that’s usually what’s the selling point. But really, it was the resistance patterns or it was a time when everyone’s very conscious about resistance. […] It was really about the benefits of choice, length, and route, and what it could bring. So, if you’re reducing morbidity and mortality. Reducing length of stay is always a good seller.

Significantly, this outcome was largely achieved through the actions of an individual clinician, who took it upon themselves to calculate the potential financial saving an AMS initiative might deliver and to “sell” the program to management using these terms. Ordinarily, the costs of AMR—and, by extension, the savings that effective AMS programs might deliver—were all but invisible. Dominant accounting practices directed executives’ attention (and, by extension, their energies and resources) toward those issues that were measured and valued within the existing reporting structures:

Participant: So, obviously from a budget point of view, there’s a huge cost associated with us ultimately not managing these patients appropriately and having to use more and more expensive drugs. From a flow perspective it’s a challenge for us around patients staying longer and needing isolation and all those sorts of things in a particularly compromised hospital, from an isolation point of view. So yeah, we don’t directly measure anything. So, it’s not front and centre in any conversation, but it is not lost on us the importance of it.

One participant speculated that change was perhaps only ever possible when those involved recognized that they stood to gain:

Participant: I think like with any change, it’s that value proposition, “What’s in it for me?” […] Some people might respond to the cost saving and the hope of reinvesting resources back into whatever is important to them. Some people will respond to the patient safety, patient value side of it. And that you’re actually doing better by your patient now, but also your patients in the future if we do it this way rather than that way. Some people will respond to the medico-legal side of things. […] It’s understanding the people you work with so that you can work out what’s important, so that you can work out how you can use that to your advantage to steer them.

At an organizational level, what “mattered” and “counted”—what the organization was structured and incentivized to respond to—were often calculations about cost and value.

In a bid to achieve budgetary ends while maintaining the deeper priorities of the organization, executives also placed considerable emphasis on better *accounting* for success within the current model of reimbursement. Described as tech efficiency below, but in other ways within other organizations, the idea here was to raise revenue through the production of data-of-success based on prevailing funding models. That is, part of the challenge was documenting activity in a way that heightened the *appearance* of “success” (as this was conceptualized within the activity-based system):

Participant: … we’ve been doing a lot on what we call tech efficiency. The better counting of the activity. Not doing more, so that we could raise more revenue, so that we can add in these services that we need that don’t necessarily fund activity.

Interviewer: Interesting.

Participant: And we’ve also been doing clinical documentation so that we have better documentation of the things we’re doing to raise more revenue … So, there’s a heap of different things we’re doing. And I think most of that stuff is a culture mindset, to be honest.

For participants who were able to play this accounting game well, activity-based funding provided the budgetary flexibility necessary for them to maximize revenue and cover additional programs:

Participant: So yeah, in some ways you’ve got more flexibility with activity-based funding [than block funding] if you can go, “Look, I can make the activity up and pay for it.” As long as you’re not already meeting your activity target, I think the government gives you some stretch funding for the next year, there can be more flexibility. […] You’ve just got to be smart about how you think about it, I suppose.

This strategy was viewed as a way to generate additional funds from existing activity, which—in addition to reducing overall budgetary pressure—could then support the non-activity that was recognized as having moral and long-term importance for the community and the organization.

### The Efficiency Imperative and the Tyranny of Success

In political realms, there are routine comparisons between household budgets and national accounts (Becker, 1962; Pollak, 2003; Wilk, 1991). Although such comparisons are routinely challenged by social, political, and economic scholars as incomparable, they were nonetheless pervasive in the interviews, reflecting an executive paradigm of cost-containment and efficiencies-as-success. Managerial prowess was very much situated between the constraints of limited means and the increased productivity of increased efficiency. Within a context of finite resources, extracting greater efficiencies through optimization was paramount. Executives drew these analogies to illustrate the need to maximize finite healthcare resourcing, illustrating the moral valance of efficiency. As one participant explained:

Participant: Here, there’s a big focus at the moment on the revenue that is generated from the activity we do, that we actually have to *live within our means*. I think all health services across Australia need to do that. There is a sustainability issue. We do need to think more clearly about what we’re doing. We do need to look at low value care and end of life care and other things and look at, is what we’re doing evidence-based and in the best interest of the patient, I suppose.

Interviewer: It’s fascinating, that comment about living within your means … And I guess my question in terms of the health service is around, how do you live within your means if the budget that you’re set doesn’t cover the burden of disease?

Participant: Well, that comes to the service provision taking priority over budget. And I’ve always maintained if the health service I’m running is providing efficient care and we can demonstrate that and we exceed the activity that’s being purchased from us and we’re in deficit as a result, then I’m okay with that … [later] if we’re doing that cost effectively and we’re in deficit, then we’re in deficit, then I’ll own that. And if they sack me over that, then so be it, because I’d go away with a clear conscience.

Ultimately, the household budget analogy provides a seemingly sensible—but, of course, deeply political—rhetorical rationalization for continued efficiencies, as opposed, for example, to higher reimbursement rates. However, what is “efficient” in any given context depends on the temporal frame of reference. Efficient delivery of acute care, for example, might be superseded by the efficiency of investment in public health interventions or a more sustainable system of funding in longer term and more multidimensional ways. And, as many of our participants pointed out, there is widespread recognition that the notions of *savings*, *deficits*, or *surpluses* derived from activity-based funding models and annualized budget cycles actually make little sense in a context of healthcare as a public good and service.

The imperative to find savings in the budget meant that funds were at times stripped out of existing programs—like AMS—in the name of efficiency, in spite of the resulting long-term inefficiencies:

Participant: So, the answer is, how do I extract those savings? How was I going to reinvest the savings, recognising that the growth in the budget does not meet the projected growth and expenditure? You either increase revenue or you extract productivity gains so that some process in microbial stewardship is automated and takes the cost, or strips the cost to some extent, out of the organisation that can be reinvested or not spent that year. So, you just pocket that saving.

While these decisions often served their budgetary aims, participants questioned whether structuring activities *reactively* around budgetary pressures was efficient in the long term:

Participant: But yeah, so it very much feels like this culture of, yeah, money.

Interviewer: Efficiency.

Participant: Yeah, efficiency. But, in a sense, it’s not efficient. You know what I mean? Like, if you take the time to actually.

Interviewer: You’d do better.

Participant: And you know that as a junior clinician, you know it as an intern or a junior nurse. You run around and run in time, but sometimes you realise if you actually take a breath for five minutes and just plan out what you need to do, you actually end up more efficient. That’s the sense at the moment. It’s just run, run and, “You have to do this, and we have to get this done, and save this money.” But it impacts on care.

As this interviewee reflected, the institutionalized imperative toward *activity*—which saw staff tasks heavily managed and fostered a valorization of busyness as a proxy for productivity—could have counterproductive effects. The momentum that gathered around existing practices made it difficult to take stock, thus contributing to an entrenched institutional inertia vis-à-vis the problem of AMR.

This issue of institutional inertia was only compounded by the temporal bounds—the annual budgets mentioned earlier—within which priorities were set. The annual basis of tight budgets that executives navigated effectively ignored issues with costs and benefits that promised to play out over the longer term. Participants noted that this tight temporal focus—and, indeed, the instability that came with planning and “achieving” on a year-by-year basis—placed significant pressure on the executives charged with setting priorities and ensuring the ongoing funding the institution needed to survive:

Participant: … you can’t escape the fact that there is politics everywhere. But it’s a case of how do you do the planning. And probably one of the biggest issues that is quite hard to manage is the way planning tends to fold around each and every financial year and perhaps little bit less longer term, if you will, because organisations may not know their budget until each financial year, and so that makes it a little bit harder. So, you’re really trying to work out then how to be efficient, how to be more efficient. And there’s always stuff you can do. It’s just a matter of how big the changes are or small the changes are and what impact they have.

Party politics and institutional politics thus intersected, as politicians and executives alike thought and acted in terms of budget periods, and faced considerable pressure to achieve visible and creditable “success” on a yearly basis.

### Organizational Performance and the Crisis of the Commons

In organizational environments driven by particular measures of performance and success, the distributed nature of responsibility for rising resistance also functioned to reduce the incentive for individual organizations to map, measure, and contain the circulation of resistance. The tendency to only measure what could be reported as a “success”—or, indeed, what the individual organization could take credit for—overlaid lackluster institutional approaches to AMR:

Participant: So, what bugs we get on the [health service region] is very dependent on the whole region’s antibiotic prescribing practices, which isn’t just what happens in the hospital. So, monitoring the patterns doesn’t necessarily give us an outcome on our performance … So, I guess by de facto, we use adherence to recommended prescribing practises. Are we sticking within best practice prescribing durations and are we sticking within dose duration, frequency, all that kind of stuff as a surrogate?

The perception that resistance was a wicked problem caused by multiple factors and actors (including some beyond the healthcare industry) further worked to justify forms of complacency and inaction. Decisionmakers posited that they were neither singularly responsible for causing, nor singularly responsible for addressing, rising resistance:

Participant: Yes, there probably is something in that [collecting resistance data in the hospital], [but] in terms of it’s a collective or shared responsibility as opposed to something we are uniquely responsible for, because you will have GP prescribers and agricultural use and that kind of stuff. I think there is something in that. I’d be interested to see the metrics and could it actually be meaningful in terms of—because that is so complex and would be so hard to gather.

In this context, comparative data—particularly data that benchmarked individual organization’s prescribing behaviors relative to other health services—was one of the few measures with the potential to impel more meaningful action. From an executive perspective, being an *outlier* in terms of behavior was a performance issue and therefore “counted”:

Participant: … I think the resistance data is really useful to look at, do we have a problem … [later] Yeah, our prescribing rates in hospitals for various antibiotics, and whether we’re out of alignment with other health services would be of real interest. And if we were out of alignment, then I’d want to understand why. And I’ve set up a stewardship program in a much leaner health service, we only had one ID physician, but we still set a system up and it worked pretty well. And I had more visibility there than I’ve got here. But there’s lots of things I haven’t got to yet in this health service. But that was of real interest to me, if we were an outlier in whatever antibiotic usage.

While benchmarking appeared to hold potential for giving comparative visibility to prescribing rates and to applying positive institutional peer-pressure toward improvements from any “outliers” below the curve, such metrified approaches to addressing the problem of AMR can quickly fall prey to “tick box” managerialism and the tyranny of micro-improvements under conditions of managerialism (Broom, Kenny, Kirby, et al., 2021).

## Discussion

We live in the era of activity, with the drive to “action” and “productivity” inflecting many aspects of social life, including the provision of healthcare. The Australian healthcare system has followed a model of activity-based funding for over a decade since the NHR Agreement was signed by federal, state, and territory governments in August 2011 (IHACPA, 2023). While the funding of health on the basis of action makes intuitive sense on one level, it gives rise to inevitable questions about what forms of “action” count and what happens within such systems when *in*activity has positive features and protective characteristic or leads to desired outcomes. These questions are both specific to AMR and universal across a range of disease threats and encompass both the structuring of care around financial models (in this case, activity-based funding) and the construction of value as implicitly connected to successful intervention (rather than, for example, the *avoidance* of intervention in the first place). Of course, the widespread conflation of activity and value has given rise to push-back and counter movements, most evident in ideas around value-based healthcare (Gray, 2017). This countermovement has included the production of new forms of metricization, including patient-reported measures (Black, 2013), in an effort to push back on the (hitherto) uncontrolled and costly march of over-interventionism. But as our executive interviews reveal, at the coalface of care, activity remains the central measure of organizational performance and value. According to the logic of activity-based funding, interventions that reduce activity—or, indeed, that result in fewer people requiring treatment—compromise the financial viability of hospital healthcare services.

The logic of activity-based funding thus poses major issues for AMR and the antimicrobial optimization agenda. Rising rates of resistant infections contribute to the complexity of almost every dimension of hospital practice. Paradoxically, however, AMR remains invisible to the organizational bottom line. With the exception of hospital-acquired resistant infections (which would fall under the banner of hospital-acquired complications), there is currently no feedback loop between negative outcomes to which AMR contributes and deleterious budgetary consequences. In fact, negative outcomes (e.g., an infection caused by an organism with documented resistance) at times attract greater financial compensation than positive outcomes (e.g., an infection caused by a fully sensitive organism) due to the increased funding for documenting the increase in complexity of treating such cases. Efforts to counteract AMR are thus not only invisible to the current models of activity-based funding but also resistant infections actually, in certain instances, increase the funding available to organizational units within health services, at least in the short term. What we thus see, here, is that the current activity-based structures of hospital financing—bound as they are to measures of volume, throughput, and activity—neither perceive nor adequately finance activities directed toward forestalling the growing threat of AMR.

Summarizing the foregoing analysis, the table below presents a schematic representation of the key interconnecting tendencies of activity-based funding across temporal, budgetary, organizational, evaluative, and processual dimensions and the ways that they contribute to the lack of attention given to AMS (Table 1).

**Table 1. Competing Imperatives of Activity-Based Funding and Antimicrobial Stewardship.** 

Tenets of Activity-Based Funding	Requirements of Antimicrobial Stewardship Programs
Short-term temporality—annual timeframe of budget-setting	AMS requires a longer scale to see impacts of actions
Budgetary priorities guide decision-making via (dollar based) cost–benefit analyses and government costs/income for health service	AMS benefits both individual and population health. But the monetary value of these benefits are difficult to estimate
Organizational units (and individual hospitals) focus on their own financial sustainability	AMS requires broad-based collaboration, and connecting the actions of one with actions and outcomes of many
Quantitative evaluative frame—only what can be measured matters/is valued in the existing reporting structures and organizational culture	AMS activities/outcomes are difficult to measure, so AMS is not valued in reports and organizational culture
Volume-driven processes—requires a focus on throughput, which can be to the detriment of a focus on prevention	AMS is oriented to curtailing and forestalling an impending problem and requires *not* doing certain tasks (not prescribing)

## Conclusion

AMR and the global push for optimization face considerable headwinds in organizational contexts where the budgetary bottom line is governed by activity. More broadly, the “wicked” nature of the problem of AMR quickly becomes an excusing condition for inaction (Fletcher, 1974; cf. Head, 2008). This study has found that activity-based funding models almost unavoidably direct attention toward volume and throughput, immediate budgetary concerns, and short-term forms of financial and governance accountability. This attention given to immediate efficiency can come at the expense of attention to long-term effectiveness or sustainability. The prioritization of short-term timeframes embedded in the funding model clashes with the kinds of longer-term coordination that is required to tackle “wicked” problems, like AMR. We have focused here on the organizational context of health services and the difficulties faced by AMS programs in such settings. But forestalling the AMR crisis is, ultimately, a shared responsibility, not a problem unique to hospitals or even health services. AMR is a crisis of the commons—and an issue of the public, long-term good—rather than one of organizational competence (Chandler, 2019). This leaves the problem of AMR largely, if not exclusively, within the realm of these executives’ sense of public purpose, what is morally right and what they *should*, rather than *can*, do.
